# Characteristics of Plasma Flow for Microwave Plasma Assisted Aerosol Deposition

**DOI:** 10.3390/nano11071705

**Published:** 2021-06-29

**Authors:** In-Je Kang, Chang-Hyun Cho, Hyonu Chang, Soo-Ouk Jang, Hyun-Jae Park, Dae-Gun Kim, Kyung-Min Lee, Ji-Hun Kim

**Affiliations:** 1 Institute of Plasma Technology, Korea Institute of Fusion Energy, Gunsan 54004, Korea; ijkang@kfe.re.kr (I.-J.K.); neojo81@kfe.re.kr (C.-H.C.); monax@kfe.re.kr (H.C.); sojang@kfe.re.kr (S.-O.J.); hyonjae@kfe.re.kr (H.-J.P.); 2 Research Center, IONES Co., Ltd., Hwaseong 18487, Korea; kdg@iones.co.kr (D.-G.K.); leekm@iones.co.kr (K.-M.L.)

**Keywords:** plasma flow velocity, Mach probe, microwave plasma source, ceramic coating

## Abstract

To validate the possibility of the developed microwave plasma source with a novel structure for plasma aerosol deposition, the characteristics of the plasma flow velocity generated from the microwave plasma source were investigated by a Mach probe with pressure variation. Simulation with the turbulent model was introduced to deduce calibration factor of the Mach probe and to compare experimental measurements for analyses of collisional plasma conditions. The results show calibration factor does not seem to be a constant parameter and highly dependent on the collision parameter. The measured plasma flow velocity, which witnessed fluctuations produced by a shock flow, was between 400 and 700 m/s. The optimized conditions for microwave plasma assisted aerosol deposition were derived by the results obtained from analyses of the parameters of microwave plasma jet. Under the optimized conditions, Y_2_O_3_ coatings deposited on an aluminum substrate were investigated using scanning electron microscope. The results presented in this study show the microwave plasma assisted aerosol deposition with the developed microwave plasma source is highly feasible for thick films with >50 μm.

## 1. Introduction

The aerosol deposition (AD) method, which is based on the impact adhesion of fine particles for the formation and micro-patterning of thick ceramic layers on substrates, is a novel coating technique for preparing thick ceramic films at room temperature [1,2,3]. In the AD method, the micro-sized ceramic particles are accelerated by gas flow in the nozzle up to a velocity of over 100 m/s and sprayed onto the substrate [1]. Moreover, no additional heating is required for the solidification of the ceramic powder [1,2]. Therefore, AD is novel and attractive coating method for ceramic thin films [1,2,3,4]. However, various limitations of AD techniques have been reported from experimental and simulation results. One notable disadvantage of AD techniques is that they have a quite low deposition efficiency, often less than 1% [5,6]. The bonding mechanism between ceramic particles has been studied, and not all factors influencing the deposition efficiency have been elucidated [5]. To produce thick films (>50 μm), the AD method has limitations, with low adhesion to the coating layer of thick films. Contaminated particles generated by plasma damage to coatings by the AD method are a major contributor to poor process reliability [7]. Advanced novel techniques with a better understanding of the AD mechanisms are required to solve various problems of the AD method for the production of high-quality thick films.

To improve the high-temperature corrosion resistance and wear resistance of materials in special environments, the technology of a direct current (DC) arc plasma spray has been widely used because it provides sufficient control over coating thickness and coating speed [8,9,10,11,12]. However, for ceramic coatings using a DC arc plasma spray, impurities from melting the electrodes cause low-quality ceramic films. The formation of pores and cracks has been observed, which decreases the mechanical properties of ceramic films deposited by a thermal arc plasma spray [9,10]. For example, Sadeghi-Fadaki reported that reductions in porosity and pore size cause higher adhesion strength when Y_2_O_3_ stabilized zirconia is used [13]. Thus, efforts need to be taken toward the fabrication of various ceramic coatings using a DC arc plasma spray to avoid the deficiencies. Although a microwave plasma source (MPS) [14] has various advantages, studies on the application of MPS to plasma aerosol deposition (PAD) are insufficient. For example, MPS generates plasma jets without any need for electrodes, which can solve problems such as impurities from melting the electrodes of a thermal arc plasma source. In addition, it produces lower flux densities than those of a DC arc plasma spray so that the powder injected in the PAD processes does not completely melt.

**Figure 1:**
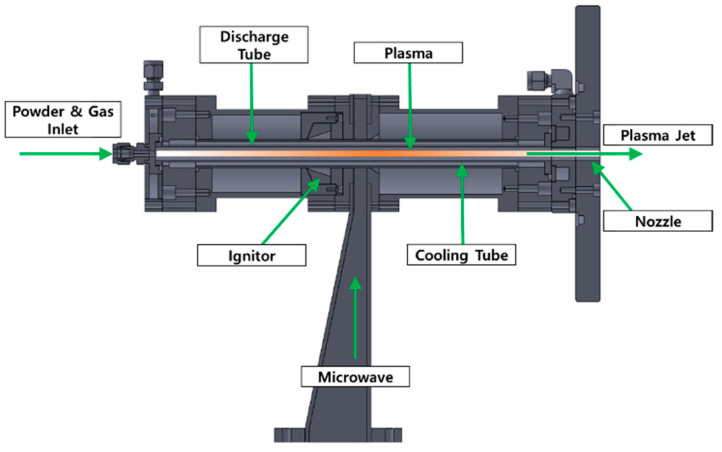
Developed microwave plasma source (MPS).

Experimental or simulation studies on flow velocity with reliable results have been lacking. However, flow velocity is one of the key parameters for the AD or PAD technique, where forming a coating by a PAD technique involves melting and accelerating particles at high velocity toward a substrate. In this study, an MPS with a novel structure for PAD was developed as a plasma generator using ignition in a surface wave discharge in a low vacuum region, which enables long-term (>4 h) operation with a stable plasma condition by introducing an effective cooling structure. This process is called “microwave plasma assisted aerosol deposition (μ-PAD)”. As shown in Figure 1, the geometric structure of the MPS was designed such that the velocity of the powder sprayed from the nozzle of a feeder could not decrease. The velocity of the powder increases as it passes through the plasma region, and the surface of the powder is melted by the high heat generated from the plasma to improve the efficiency of the μ-PAD. In other words, melting only the surface of the powder using microwave plasma is the key to the concept of μ-PAD.

To validate the feasibility of the developed MPS for μ-PAD, the characteristics of the plasma flow velocity generated from the MPS were investigated. We adopted a Mach probe (MP) [15] with the simulation of a turbulent model for the analysis of plasma flow velocity in collisional microwave plasma jets. In addition, using μ-PAD, with the optimized conditions of the MPS, Y_2_O_3_ coatings were deposited on an aluminum substrate, and the Y_2_O_3_ coating layer was investigated by scanning electron microscope (SEM).

## 2. Experimental Setup

**Figure 2:**
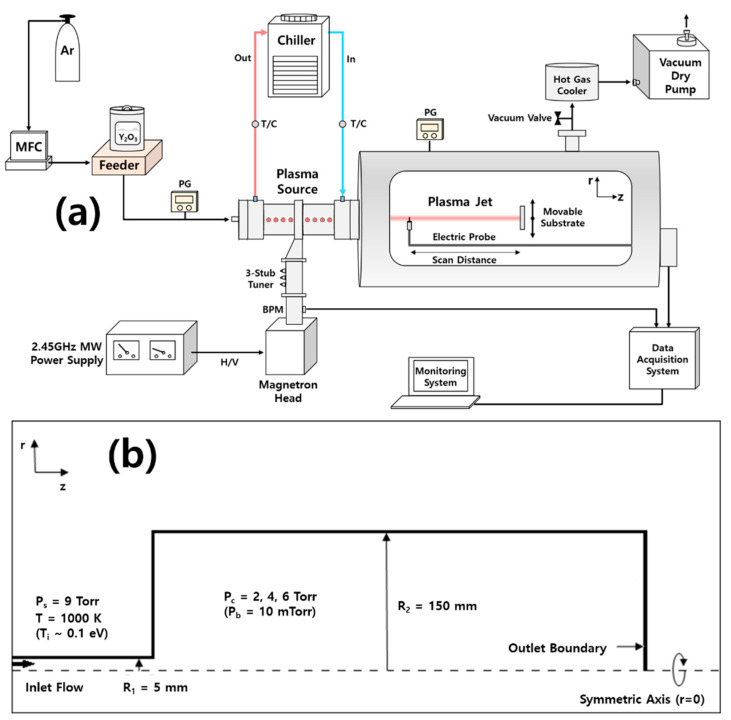
(**a**) Experimental setup for the measurement of plasma flow velocity generated from the developed microwave plasma source (MPS) for microwave plasma assisted aerosol deposition (μ-PAD). (**b**) Schematic diagram for plasma flow measurement and simulation. MFC: mass flow controller, PG: pressure gauge, TC: thermal couple, BPM: bidirectional power meter, MW: microwave, P_s_: source pressure, P_c_: operating chamber pressure, P_b_: base chamber pressure, R_1_: source radius and R_2_: chamber radius.

**Figure 3:**
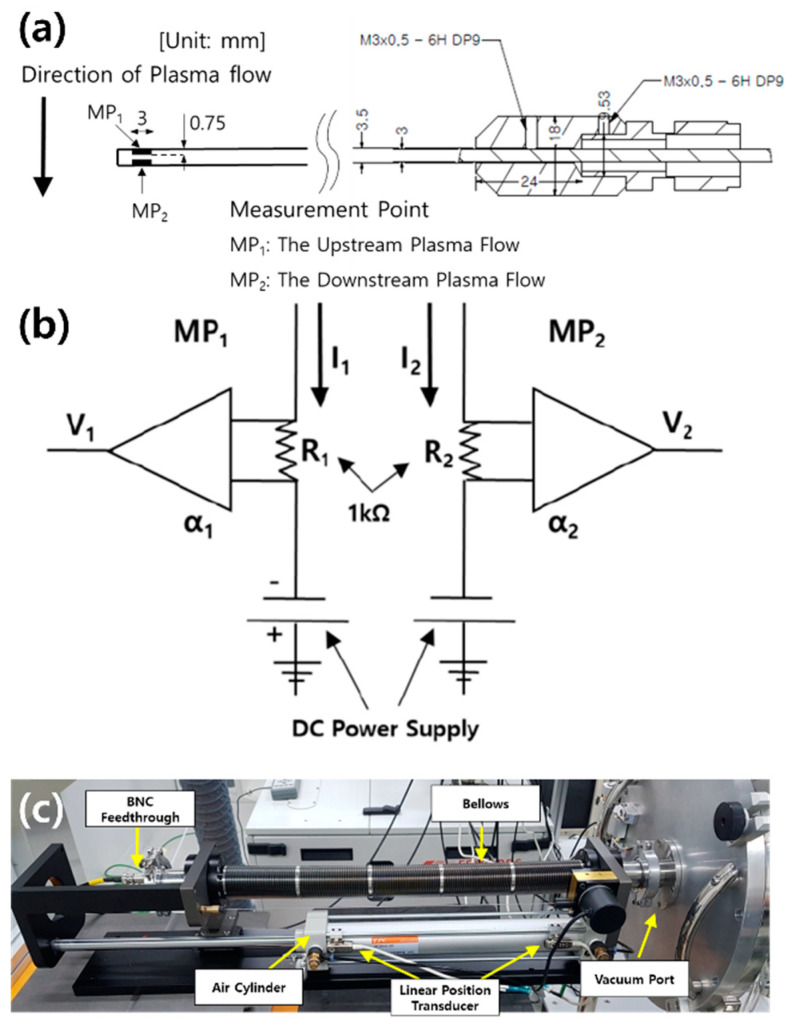
(**a**) Geometry and (**b**) circuit of the Mach probe (MP) and (**c**) the scanning system for MP. Area of probe tip = 2.25 × 10^−2^ cm^2^. MP_1_ and MP_2_ are for measurement of upstream and downstream ion saturation current densities, respectively. Measured voltage from each probe is $V_{1}=\alpha_{1}I_{1}R_{1}$ for upstream current (MP_1_) and $V_{2}=\alpha_{2}I_{2}R_{2}$ for downstream current (MP_2_), where α, *I*, and *R* are conversion factors, ion saturation current and resistors, respectively.

Figure 2 shows a simplified schematic view of the experimental setup for the measurement of the plasma flow velocity generated from the developed MPS for μ-PAD. The cylindrical vacuum chamber had a diameter of 380 mm and an axial length of 720 cm. The nozzle diameter of the MPS was 1 cm, and argon gases with Y_2_O_3_ powder, injected by a feeder, were sprayed by the nozzle of the MPS. The plasma was generated using a 2.45 GHz microwave with 1–1.5 kW power, when the reflected power was reduced by <0.1% using a 3-stub tuner with a bidirectional power meter (BPM). A power supply, operated at 15 kV, 30 mA, and 25 kHz, was used for the ignition of the MPS. Argon was used as the working gas. It was injected from the rear port of the MPS with Y_2_O_3_ powders, and a mass flow controller (MFC) maintained a constant gas flow rate of 10 slm. In this study, Y_2_O_3_ powder was not injected when measuring plasma flow velocity by an MP, and the Y_2_O_3_ coatings were deposited on an aluminum substrate by μ-PAD after optimizing the plasma jet generated by MPS. The base pressure was 10 mTorr, and the operating pressure was 9 Torr in the MPS and 2–6 Torr in the vacuum chamber, controlled by a vacuum valve between a vacuum chamber and a hot gas cooler, for argon gas, which is 10 slm. The z-axial plasma flow profiles were measured by the parallel MP, which consisted of two tungsten tips and a ceramic insulator between the tips. The geometry of the MP, which has a collective area (A) of probe tips (2.25 × 10^−2^ cm^2^) and a simple circuit, is shown in Figure 3a,b. Using bipolar operational power, a negative bias (−100 V) was applied to probe tips for the collection of ion saturation currents (*I_sat_*). For MP data, the measured voltage from each probe is given as $V_{1}=\alpha_{1}I_{1}R_{1}$ for the upstream probe (MP_1_) and $V_{2}=\alpha_{2}I_{2}R_{2}$ for the downstream probe (MP_2_), where α, *I*, and *R* are the conversion factor, ion saturation current, and resistance (1 kΩ), respectively. To reduce the uncertainty of data acquisition, α is introduced for calibration of the tip area and circuit differences with the BNC cable and two power supplies.

**Figure 4:**
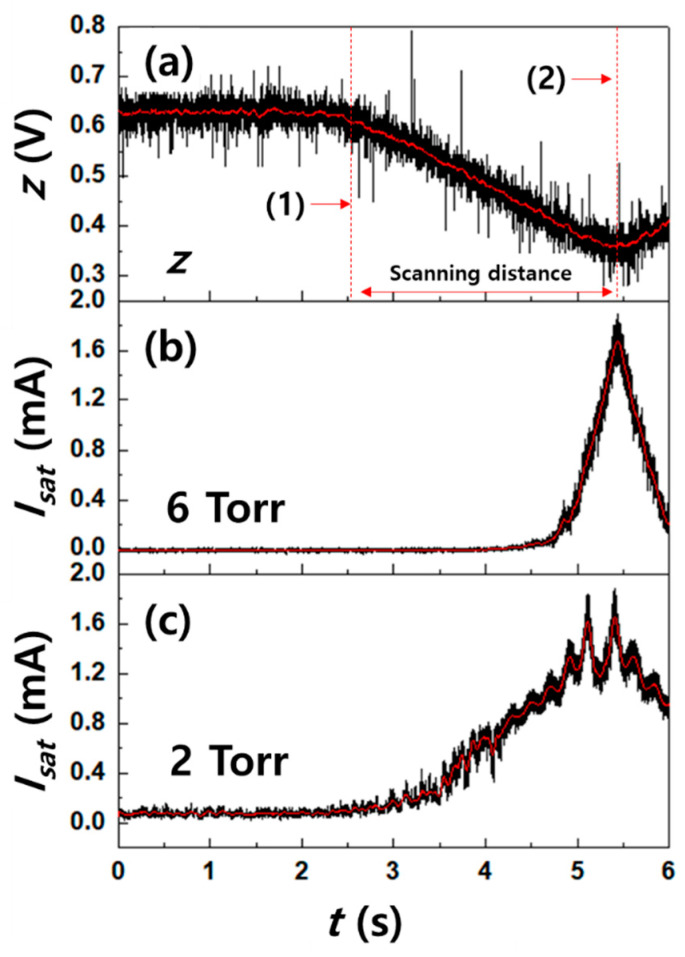
Raw data for (**a**) the voltage measured by a linear position transducer for z-axial position (z), (**b**) ion saturation current at microwave power = 1 kW and base pressure = 6 Torr and (**c**) ion saturation current at microwave power = 1 kW and base pressure = 2 Torr, obtained from performance test of a scanning system for the Mach probe. z-axial position: (1) = 300 ± 2.5 mm and (2) = 10 ± 2.5 mm from the nozzle exit of a microwave plasma.

As shown in Figure 3c, to investigate the effect of the plasma flow velocity on the operating pressure, a scanning system with electric probe tips was used, which can scan the z-axial plasma profiles with a scan speed 0.1 m/s. The z-axial position was converted from the voltage signals measured using linear position transducers. Figure 4 shows the raw data of MP for voltages of z-axial position (z) and *I_sat_*, obtained from the performance test of a scanning system.

## 3. Measurement of Plasma Flow

When an MP is composed of two separate directional probes with strongly negative biased potential, one collects the current density ($J_{up}$) with the upstream plasma flow by MP_1_, and the other collects current density ($J_{dn}$) moving against the downstream plasma flow by MP_2_ (refer to Figure 3) [15]. Owing to plasma flow, these two currents show asymmetry, producing a measured ratio (*R_m_*) of current densities that is greater than one: $R_{m}=J_{up}/J_{dn}\geq 1$ [16,17]. For dimensional analysis of a Mach probe with ratio of current densities reported by Chung [15,17], the one-dimensional continuity equation for ions is described as:

(1) $$\frac{\partial n_{i}}{\partial t}+\frac{\partial }{\partial x}n_{i}v=0$$

which leads to

(2) $$\frac{\partial n_{i}}{\partial t}n_{i}v=-\frac{\partial n_{i}}{\partial t}$$

where $n_{i}$, v, and x are the ion density, plasma flow velocity, and the coordinate for the direction of the flow or magnetic field, respectively. Here, $\partial n_{i}/\partial t$ can be treated as a source term, which can also be obtained from the steady-state two-dimensional continuity equation, which is the same as those in most fluid models, i.e., $-\partial \left(n_{i}v\right)/\partial y\;$~$-\partial n_{i}/\partial t$ in dimensional analysis, where y is the coordinate for the perpendicular direction to the flow or the magnetic field. Equation (2) can be rearranged as

(3) $$\partial J=-\frac{dx}{dt}\partial n=-v\partial n_{i}$$

where $J\equiv n_{i}v$. Then from the momentum equation, one can determine the following with the Boltzmann electrons:

(4) $$m_{i}n_{i}\frac{dv}{dt}=\frac{dP_{i}}{dx}+en_{i}E=-\left(T_{i}+T_{e}\right)\frac{dn_{i}}{dx}\equiv -T\frac{dn_{i}}{dx}$$

where $m_{i}$, e, $P_{i}$, E, $T_{i}$, and $T_{e}$ are the ion mass, electron charge, ion pressure, electric field intensity, ion and electron temperature, respectively. By multiplying both of the terms of Equations (3) and (4) by dx and using v=dx/dt, it becomes

(5) $$Jdv=-c_{s}^{2}dn_{i}=\frac{c_{s}^{2}}{v}dJ$$

The separation of J and v leads to

(6) $$d\frac{v^{2}}{2c_{s}^{2}}=d\left(\ln J\right),$$

which becomes

(7) $$J=J_{0}\exp\left[\frac{v^{2}}{2c_{s}^{2}}\right]=J_{0}\exp\left[\frac{M^{2}}{2}\right]$$

where $M\equiv \frac{v}{c_{s}}=v/\sqrt{\left(T_{e}+T_{i}\right)/m_{i}}$, $c_{s}$ and $J_{0}$ are Bohm velocity and the unperturbed ion flux. Since drift flow affects the flow in perturbation regions, one can assume $M_{up}\approx M\left(x\right)+\beta M_{\infty }$ and $M_{dn}\approx M\left(x\right)-\beta M_{\infty }$, where M(x) and $M_{\infty }$ are a normalized flow velocity of the perturbation region without drift flow and a normalized flow velocity, respectively. Also, β is the constant reflecting the effects of magnetic field, collisionality, viscosity, ion temperature, and other factors. Then

(8) $$R_{m}\equiv \frac{J_{up}}{J_{dn}}=\exp\left[\frac{M_{up}^{2}-M_{dn}^{2}}{2}\right]=\exp\left[M\left(x\right)\cdot 2\beta M_{\infty }\right]=\exp\left[k_{f}M_{\infty }\right]$$

where $k_{f}=2\beta M\left(x\right)$, which is calibration factor of a Mach probe.

For the analysis of the experimental Mach probe data on plasma flow velocity by theories of ion collection [15,16,17], the ratio of the upstream to downstream current densities is given as

(9) $$R_{m}=\frac{J_{up}}{J_{dn}}=\frac{I_{1}/A_{1}}{I_{2}/A_{2}}=\frac{V_{1}/\alpha_{1}R_{1}A_{1}}{V_{2}/\alpha_{2}R_{2}A_{2}}$$

**Figure 5:**
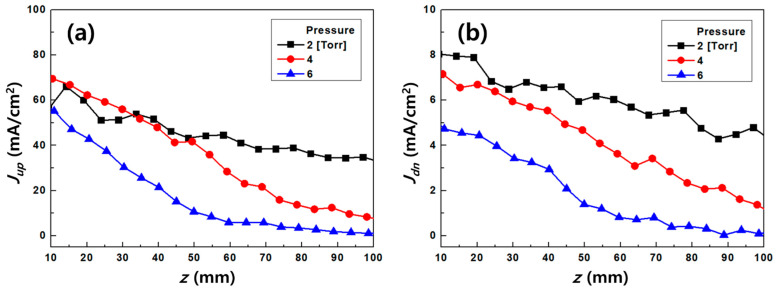
Current densities (*J*) of the Mach probe: (**a**) upstream and (**b**) downstream for base pressure variation at 1 kW microwave power.

The measured voltages from each probe, along with the conversion factors, resistance values and collective area, were used to calculate $M_{\infty }$. Figure 5 and Table 1 show the results of the current densities calculated using Equation (9) with pressure variation, which was used to calculate the ratio of upstream to downstream current densities. Using various models [15,16,17,18,19,20,21] with Equations (8) and (9) for the plasma flow velocity, $M_{\infty }$ was defined as

(10) $$M_{\infty }=\frac{v}{c_{s}}=\frac{1}{K_{f}}\ln\left[R_{m}\right]$$

*T_e_* ~ 1 eV was measured by a Langmuir probe, and *T_i_* = 0.1*T_e_* was assumed for the calculation of $c_{s}=\sqrt{\left(T_{e}+T_{i}\right)/m_{i}}$. The difference in $c_{s}$ between the approximation condition (*T_e_* ~ 1 eV) and the measured results (*T_e_* = 0.92–1.09 eV) as a function of pressure (2–6 Torr) was less than 5%. Therefore, the fixed condition (*T_e_* = 1 eV) was used for the Bohm velocity (1629 m/s). The calibration factors for collisionless conditions have been studied using various kinetic and fluid models [19,20,21]. However, the overestimation of plasma flow velocity in collisional plasmas measured by an MP has been reported because of the effect of ion-neutral collision [22,23,24]. In collisional plasma, the overall flow behavior can be described by the conventional gas dynamic theory, because the major constituent of the plasma is still neutral atoms due to the low degree of ionization in typical plasma sources in collisional conditions [25,26]. To consider this issue, plasma velocity ≈ neutral gas flow velocity (u) was assumed and the turbulent model was introduced to deduce *K_f_* for collisional conditions.

## 4. Simulation Model

The model studies were also involved in capturing the measured oscillatory phenomena of turbulent high-velocity flow in collisional plasmas. The *High Mach Number Flow Module* contained in the commercial software COMSOL Multiphysics (V. 5.3a, COMSOL Multiphysics, Stockholm, Sweden) was utilized [27]. The governing equations for this model are the Navier–Stokes equations in the stationary state (du/dt=0), which are composed of equations for continuity and momentum conservation. The equation for energy conservation is simultaneously correlated with the equations and their expressions are as follows:

(11) (continuity)  ∇·(ρu)=0

(12) $$(\mathrm{momentum})\;\;\rho \left(u\cdot \nabla \right)u=\nabla \cdot \left[-pI+\left(\mu +\mu_{T}\right)\left(\nabla u+{\left(\nabla u\right)}^{T}\right)-\frac{2}{3}\left(\mu +\mu_{T}\right)\left(\nabla \cdot u\right)I-\frac{2}{3}\rho kI\right]$$

(13) $$(\mathrm{energy}\;\mathrm{conservation})\;\;\rho C_{p}u\cdot \nabla T-k_{t}\nabla^{2}T=Q$$

where ρ, u, p, μ, $\mu_{T}$, $C_{p}$, $k_{t}$ T, and Q are the gas density, gas velocity, pressure, dynamic viscosity, turbulent dynamic viscosity, heat capacity at constant pressure, thermal conductivity, gas temperature, and heat source, respectively. For the simulation of the turbulent model, the Reynolds-averaged Navier–Stokes (RANS) model is used, which includes the *k*–*ε* model. Each of *k*–equation for turbulent kinetic energy (*k*) and *ε*-equation for the turbulent dissipation rate (*ε*) is

(14) $$\rho \left(u\cdot \nabla \right)k=\nabla \cdot \left[\left(\mu +\frac{\mu_{T}}{\sigma_{k}}\right)\nabla k\right]+p_{k}-\rho \varepsilon$$

and

(15) $$\rho \left(u\cdot \nabla \right)\varepsilon =\nabla \cdot \left[\left(\mu +\frac{\mu_{T}}{\sigma_{\varepsilon }}\right)\nabla \varepsilon \right]+C_{\varepsilon 1}\frac{\varepsilon }{k}p_{k}-C_{\varepsilon 2}\rho \frac{\varepsilon^{2}}{k}$$

where

(16) $$\mu_{T}=\rho C_{\mu }\frac{k^{2}}{\varepsilon }$$

(17) $$p_{k}=\mu_{T}\left[\nabla u+{\left(\nabla u\right)}^{T}-\frac{2}{3}{\left(\nabla \cdot u\right)}^{2}\right]-\frac{2}{3}\rho k\nabla \cdot u$$

The free constants for these equations are set to $C_{\varepsilon 1}=1.44$, $C_{\varepsilon 2}=1.92$, $C_{\mu }=0.09$, $\sigma_{k}=1$, and $\sigma_{\varepsilon }=1.3$, which were validated by numerous iterations of data fitting for a wide range of turbulent flows [28]. The dynamic viscosity μ and thermal conductivity $k_{t}$ are determined by Sutherland’s law, which relates the quantities to the gas temperature and constants dependent on the gas species. Sutherland’s laws are as expressed as follows:

(18) $$\frac{\mu }{\mu_{0}}={\left(\frac{T}{T_{0}}\right)}^{3/2}\frac{T_{0}+S_{\mu }}{T+S_{\mu }}$$

(19) $$\frac{k}{k_{0}}={\left(\frac{T}{T_{0}}\right)}^{3/2}\frac{T_{0}+S_{k}}{T+S_{k}}$$

where $\mu_{0}=2.125\times 10^{-5}\;N\cdot s/m^{2}$, $T_{0}=273\;K$, $S_{\mu }=114\;K$, $k_{0}=0.0163\;W/\left(m\cdot K\right)$, $S_{k}=170\;K$, and the values are those of argon species [29].

The geometry of the MPS was reflected in two dimensional axisymmetric simulations, and the simulation domain was decomposed into 61,532 meshes. A schematic diagram is shown in Figure 2b. The argon flow, which has a Mach number of 1.5 and a temperature of 1000 K (*T_i_* ~ 0.1 eV), is heated by a microwave power of 1 kW, passing through a tube, with a diameter of 10 mm, and spurted out of the chamber with a diameter of 200 mm. The base pressure in the MPS was set to 10 mTorr and the pressure on the inlet boundary (in MPS) was set to 9 Torr based on the experimentally measured result. To estimate $K_{f}$ and compare the experimental measurements, the gas flow velocities were investigated at different chamber pressures: 2, 4, and 6 Torr.

## 5. Results and Discussion

**Figure 6:**
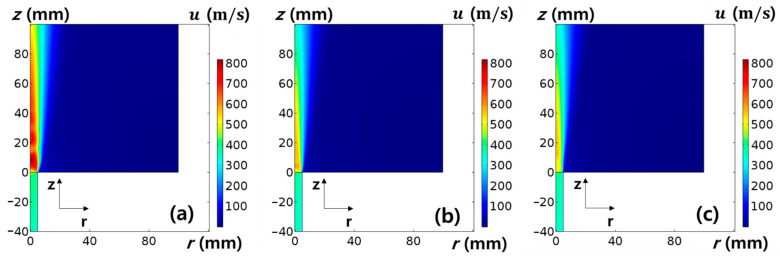
Simulation results of gas flow velocity (*u*) along the z-axis. Base (chamber) pressure variation at the fixed microwave power = 1 kW. (**a**) 2 Torr, (**b**) 4 Torr, and (**c**) 6 Torr. z = 0 is the nozzle exit of a microwave plasma source.

**Figure 7:**
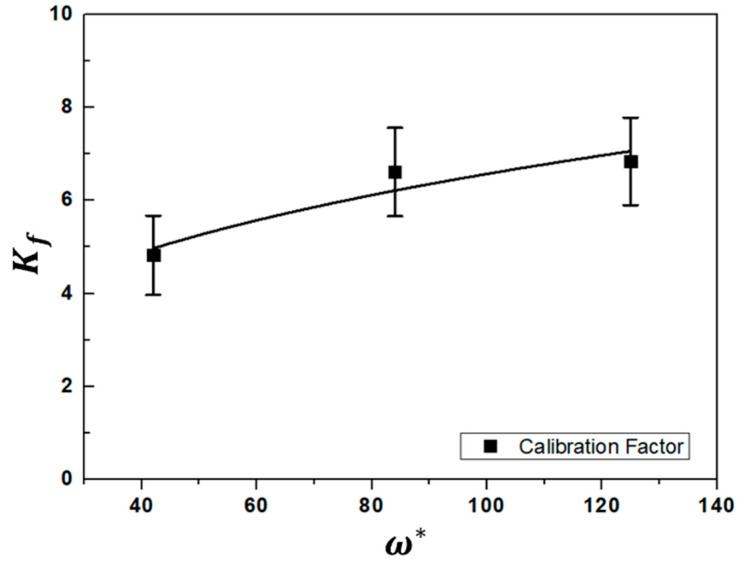
Results for the effect of normalized ionization collision frequency ($\omega^{*}$) on the calibration factor (*K_f_*).

Using the 2D simulation results of u at the center of the z-axis with pressure variation, as shown in Figure 6, the calibration factor was calculated using Equation (10) as $K_{f}=c_{s}u^{-1}\ln\left[R_{m}\right]$. Figure 7 shows the results for the relation between the calibration factor and normalized ionization collision frequency ($\omega^{*}\equiv \omega a/c_{s}$) as a dimensionless parameter to deduce the effect of the collision parameter on the calibration factor, where ω and a are the ionization collision frequency and the probe radius [15,23,24]. The calibration factor does not seem to be a constant parameter at 40 < $\omega^{*}$ < 130 and highly dependent on the collision parameters. In addition, the calculated calibration factor for collisional plasma conditions is much higher than that for collisionless models [15], which have similar results for the overestimation of plasma flow velocity in collisional plasmas by an MP [23,24].

**Figure 8:**
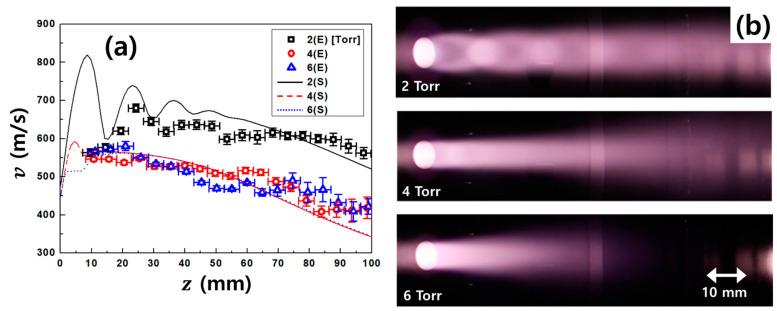
(**a**) Results of the plasma flow velocity (v) along the z-axis measured by the Mach probe and (**b**) images of plasma jets generated from the microwave plasma source (MPS). Base (chamber) pressure variation at the fixed microwave power = 1 kW. Note that z at the x-axis is the position from the nozzle exit (z = 0) of the microwave plasma source. (E) and (S) in this figure legend are experiment and simulation results, respectively.

**Figure 9:**
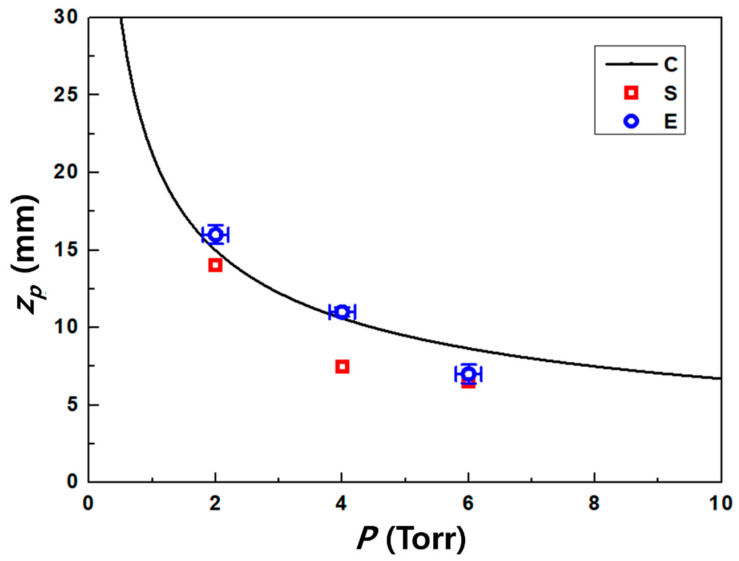
Results for the first position of the normal shock. The legend indicates that C = calculation by Equation (20), S = simulation, E = experiments by the Mach probe and camera images.

Figure 8 and Table 2 show the plasma flow velocity along the z-axis measured by an MP and camera images of microwave plasma jets for operating chamber pressure variation at a fixed microwave power of 1 kW. The experimental results of the plasma flow velocity were found to be in good agreement with the simulation results. The measured plasma flow velocity was between 400 and 700 m/s, and it gradually decreased along the z-axis. The maximum value was ~700 m/s when the experimental conditions were z = 25 mm, microwave power = 1 kW, and operating chamber pressure = 2 Torr. At 2 Torr, the plasma jet was over-expanded with the formation of oblique shocks at the edge of the nozzle exit, converging to the jet axis. On the other hand, it was not clearly observed at 4 and 6 Torr as higher pressures. The results show similar tendencies with the phenomena for supersonic flow in arc plasma jets, experimentally reported by Namba [26] and Gindrat [30]. The position of the normal shock front varies with the flow and background pressure as predicted by

(20) $$z_{p}=0.67\times d\sqrt{\frac{P_{s}}{P_{c}}}$$

where *d*, *P_s_* and *P_c_* are the source nozzle diameter, source pressure at the nozzle throat, and chamber pressure [25,26,31]. Further, d=10 mm, $P_{s}=9$ Torr, and $P_{c}=2-6$ Torr were used for the calculation of $z_{p}$ (refer to Figure 2). The results calculated using Equation (20) for $z_{p}$ were compared with the results of the simulation and experiment obtained using MP and camera images. Figure 9 shows the first positions of the normal shock for the operating pressure variation. The simulation and experiment results, which can be fitted in the form calculated by Equation (20), were in good agreement for the estimation of $z_{p}$ with little uncertainty in absolute values.

**Figure 10:**
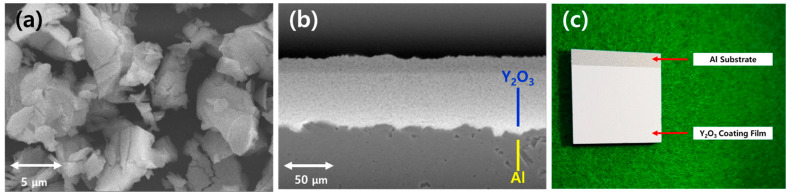
Images from the scanning electron microscope (SEM) for (**a**) the Y_2_O_3_ raw powder material and (**b**) Y_2_O_3_ coating layer and (**c**) image of the Y_2_O_3_ coating sample deposited by the microwave plasma source (MPS).

After the optimization of conditions considering the substrate position, plasma flow velocity, etc. of MPS for μ-PAD were derived from the results obtained from the simulation and experiment for the analyses of the parameters of the microwave plasma jet, the Y_2_O_3_ coatings were deposited on an aluminum substrate. Y_2_O_3_ powder was injected at 4 g/min with a particle size of ~5 μm, which was a commercial Y_2_O_3_ powder with 99% purity as shown in Figure 10a. Considering the window of deposition as a function of the average particle diameter and velocity for intrinsically brittle materials from molecular dynamics simulations in comparison to the analytical fracture model [6], the μ-PAD was conducted under experimental conditions with the highest plasma velocity. The particles with excessive impact velocity, rather than the bonding between the particles themselves, contribute to the erosion of the coating layer [5]. However, this was not observed in this study because the particle velocity generated from the nozzle of the MPS was expected to be much lower than the measured plasma flow velocity owing to the increased collision frequency and the mass ratio of Y_2_O_3_ particles and gases. Mounting the substrate at a position with higher plasma velocity, where the distance between the nozzle and the substrate was less than 50 mm, resulted in negative effects, such as a changed μ-PAD condition and low quality of coating layers, due to the accumulation of Y_2_O_3_ particles at the nozzle or damage to the nozzle by the Y_2_O_3_ particles backscattered from the substrate and the fluctuation of plasma parameters produced by the shock flow. Therefore, the distance between the nozzle and the substrate was fixed at 50 mm, which was the position for plasma flow velocity of 600 < v < 650 m/s under the experimental conditions of the MPS for the Y_2_O_3_ coatings: microwave power = 1 kW and operating pressure = 2 Torr at 10 slm in the vacuum chamber. This condition results in a relatively lower fluctuation of plasmas formed from the shock flow, as shown in the results on the characteristics of the plasma flow. The substrate was scanned repeatedly at a speed of 100 mm/s. Figure 10b,c show the results of Y_2_O_3_ coatings on an aluminum substrate for 10 min, which were analyzed by SEM for the microstructure with a cross-sectional view. The thickness of the coating layer was ~50 μm, with pore properties <10%.

In future work, the coating efficiencies, hardness, surface roughness and pore properties of Y_2_O_3_ coatings, deposited on various substrates, will be studied with SEM, X-ray diffraction (XRD), Fourier Transform Infrared Spectroscopy (FTIR), etc.

## 6. Summary and Conclusions

An MPS with a novel structure was developed as a plasma generator for μ-PAD. To validate the potential of the developed MPS for μ-PAD, the characteristics of the plasma flow velocity generated from the MPS were investigated using an MP with pressure variation. Simulation with the turbulent model was introduced to deduce the calibration factors of the Mach probe and compare experimental measurements for analyses of collisional plasma conditions, owing to the overestimation of plasma flow velocity in collisional plasmas measured by an MP with collisionless models. The results show that the calibration factor does not seem to be a constant parameter and is highly dependent on the collision parameter. In addition, the calculated calibration factor of 4 < *K_f_* < 7 under collisional plasma conditions (40 < $\omega^{*}$ < 125) is much higher than that of collisionless models. The measured plasma flow velocity, which witnessed fluctuations produced by a shock flow, was between 400 and 700 m/s. The optimized conditions considering substrate position, plasma flow velocity, etc. of the developed MPS for μ-PAD were derived by using the results obtained from the simulation and experiment for analyses on the parameters of the microwave plasma jet. The optimized conditions were as follows: substrate position (z) = 50 mm and plasma flow velocity = 600 < v < 650 m/s at a microwave power of 1 kW and operating pressure of 2 Torr at an argon gas flow rate of 10 slm in the vacuum chamber. Under the optimized conditions, Y_2_O_3_ coatings deposited on an aluminum substrate were investigated using SEM. After applying the μ-PAD for 10 min., a coating layer thickness ~50 μm and pore properties of <10% were observed. By comparison with AD results of ≤10 μm, the results presented in this study show that the μ-PAD with the developed MPS is a highly feasible as an alternative to AD with various limitations, such as low adhesion for the coating layer of thick films.

This study contributes to the understanding of a Mach probe for the measurement of plasma flow velocity in highly collisional conditions. In addition, the measured physical properties or tendencies of the plasma jets will serve dry processes for the synthesis of nanomaterials with plasma sources such as deposition or etching in semiconductor fields, because the surface damage to parts or walls of semiconductor equipment which require the coated ceramic materials with high plasma resistance has emerged as a new issue due to etching through the corrosive ionized gases or the extreme ultraviolet used.

## Figures and Tables

**Table 1:** Measured results of current densities for the Mach probe. The *J* has average values for z = ±2.5 mm with the standard deviation (≤5%).

*z* (mm)	2 (Torr)			4 (Torr)			6 (Torr)		
	*J_up_* (mA/cm^2^)	*J_dn_* (mA/cm^2^)	ln(*R_m_*)	*J_up_* (mA/cm^2^)	*J_dn_* (mA/cm^2^)	ln(*R_m_*)	*J_up_* (mA/cm^2^)	*J_dn_* (mA/cm^2^)	ln(*R_m_*)
10	58.16	8.05	1.98	69.56	7.13	2.28	56.23	4.87	2.45
20	57.73	7.81	2.00	62.14	6.69	2.23	43.30	4.52	2.26
30	52.27	6.43	2.10	55.65	5.97	2.23	30.63	3.47	2.18
40	49.98	6.68	2.01	47.28	5.49	2.15	21.47	2.94	1.99
50	43.51	6.00	1.98	40.83	4.55	2.19	10.56	1.37	2.04
60	43.55	5.75	2.02	25.43	3.28	2.05	5.63	0.80	1.95
70	38.06	5.38	1.96	20.51	3.24	1.85	5.53	0.75	2.00
80	37.93	5.37	1.95	12.61	2.20	1.75	3.44	0.42	2.11
90	34.32	4.45	2.04	11.90	2.05	1.76	1.69	0.11	2.72
100	33.91	4.55	2.01	7.74	1.31	1.78	1.19	0.20	1.81

**Table 2:** Comparison between experiment (E) and simulation (S) results for the plasma flow velocity along the z-axis. The E has average values for *z* = ±2.5 mm.

z (mm)	2 (Torr)		4 (Torr)		6 (Torr)	
	E (m/s)	S (m/s)	E (m/s)	S (m/s)	E (m/s)	S (m/s)
10	563 ± 10	778	546 ± 05	568	566 ± 05	552
20	620 ± 09	711	537 ± 06	563	580 ± 12	561
30	652 ± 11	656	527 ± 06	554	533 ± 05	554
40	635 ± 12	681	529 ± 07	540	514 ± 05	540
50	632 ± 14	668	509 ± 08	514	469 ± 07	516
60	609 ± 16	645	516 ± 08	479	484 ± 07	482
70	615 ± 09	621	487 ± 10	445	465 ± 15	448
80	607 ± 11	589	437 ± 15	407	459 ± 27	410
90	597 ± 16	553	414 ± 20	371	432 ± 25	374
100	562 ± 14	520	418 ± 26	342	420 ± 23	345

## Data Availability

The data presented in this study are available on request from the first or corresponding author.
